# On-Campus vs Distance Tutorials in Preparatory Courses for Mathematics Student Teachers – Performance Gains and Influencing Factors

**DOI:** 10.1007/s40753-023-00221-3

**Published:** 2023-06-13

**Authors:** Katharina Kirsten, Gilbert Greefrath

**Affiliations:** Institute of Mathematics Education and Computer Science Education, University of Münster, Münster, Germany

**Keywords:** Preparatory course, Transition from school to university, Course modality, Affective and cognitive dispositions, Person-environment fit, Innovative teaching and learning practices

## Abstract

Due to COVID-19 pandemic restrictions, new instructional designs for mathematics courses have recently been developed. Unlike traditional e-learning courses, distance learning via videoconferencing contains more synchronous elements and is therefore more closely related to classroom instruction. Since theories of person-environment fit suggest that course modality may have an impact on learning processes, this study compares the effectiveness of on-campus (in-person) and synchronous distance tutorials as essential components of a mathematics preparatory course. Using a within-between-subject design, we examined performance gains of first-year student teachers (primary and lower secondary level) during a two-week preparatory course in relation to (1) tutorial variation, (2) students’ prior knowledge, and (3) general and specific affective factors. Overall, our results indicate that preparatory courses with on-campus and distance tutorials can be similarly effective. However, considering students’ prior knowledge as measured by an entrance test, the course variant proved to be a decisive factor for students with higher test scores: While students with lower scores showed comparable performance gains in both on-campus and distance tutorials, students with higher scores increased their mathematics performance only in on-campus tutorials. Furthermore, the results indicate that the impact of affective factors on course performance differs in the two tutorial variants. While students’ self-efficacy and engagement predict learning outcomes in on-campus tutorials, mathematics performance in distance tutorials is positively influenced by self-efficacy and satisfaction and negatively influenced by procrastination and social relatedness. Thus, the results shed light on how instructors can design on-campus and distance tutorials to promote effective learning.

## Introduction

In the German tradition, many universities offer non-credit preparatory courses to first-year students aimed at strengthening the content-related and affective prerequisites for studies involving mathematics. Such courses are offered to all entering students on a voluntary basis and take place as a summer-school-like two- to six-week event before the first semester begins (Büchele, 2020; Eichler & Gradwohl, 2021). If participation in preparatory courses is voluntary, courses should be designed to appeal to students and encourage them to participate (Büchele et al., 2022; Park et al., 2018). In this context, the question of an appropriate course design has been discussed for several years, focusing in particular on the course modality (Biehler et al., 2018). For example, at some universities, preparatory courses have been offered as e-learning courses for many years to accommodate working professionals or different paces of learning (Dondorf et al., 2016; Greenberg & Williams, 2008). Other digital course designs have recently been tested. Prompted by the COVID-19 pandemic, courses were offered in many places via videoconferencing systems. In contrast to traditional e-learning courses, these distance courses are often more closely orientated to on-campus teaching and therefore contain more synchronous elements (Büchele et al., 2021; Radmehr & Goodchild, 2022). However, compared with on-campus courses, distance learning is considered more demanding, since it implicitly requires a high level of self-regulation and technical capabilities (Artino & Stephens, 2009; Michinov et al., 2011; Reinhold et al., 2021). Therefore, it is questionable whether these new distance courses are suitable for achieving course-related goals. To fill this research gap, this study investigates the benefits of newer distance courses compared to traditional on-campus courses. Using the example of a two-week preparatory course for student teachers at a German university, we therefore examined the students’ performance gains in relation to course modality, students’ prior knowledge, and general and specific affective factors.

## Preparatory Courses in Mathematics-Related Study Programs

Preparatory courses are associated with different course names (e.g. compensatory, bridging courses), often with different contexts and goals. The common goal of these courses, however, is to create a common ground on which first-year university mathematics courses can build. Concerning the institutional structure, Higbee et al. (2005) distinguish between prerequisite acquisition models, in which students who feel unprepared for university learning work on improving their mathematical skills prior to taking a credit-bearing course, and concurrent acquisition, in which students take an additional course during the semester and thus concurrently with the regular mathematics course. Participation in both types of courses may be mandatory, meaning dependent on placement on a centralized test, or voluntary, meaning dependent on student self-assessment (Büchele et al., 2022; Park et al., 2018). In this paper, we focus on voluntary preparatory courses based on the prerequisite acquisition model. This type of preparatory course corresponds to the common practice in Germany and is also widespread in other European countries (Biehler et al., 2018; Büchele et al., 2022).

### Preparatory Courses in Germany

Since students in Germany choose their major at the very beginning of their studies, the topics covered in the preparatory course may vary depending on the corresponding study program (e.g. STEM education, mathematics majors, teacher education). However, there is a common ground to cover both cognitive and affective aspects of study prerequisites (Biehler et al., 2018; Hochmuth et al., 2018). Therefore, the contents of a preparatory course are often divided into two parts: On the one hand, topics of school mathematics are repeated, on the other hand, first topics of university mathematics are taken up in order to address differences between school and university mathematics and to prepare mathematical thinking and writing at undergraduate university level (Deeken et al., 2020). Thus, preparatory courses in Germany are aimed not only at first-year students who want to reinforce particular concepts from school mathematics but also at those who want to prepare themselves for learning at university level.

Nevertheless, the repetition of school knowledge remains a key component and is growing in importance as many universities increasingly report knowledge gaps among their students. In fact, entrance tests, which some universities conduct at the beginning of studies, reveal a large proportion of students faced challenges, even though the tests primarily ask about secondary school knowledge (Bach et al., 2018; Greefrath et al., 2017). Participation in preparatory courses is therefore strongly recommended but remains voluntary.

Like regular, credit-bearing mathematics courses, preparatory courses are generally held as a lecture with supplementary tutorials (Biehler et al., 2018). While the lectures are for the whole group, the tutorials take place in small groups and offer opportunities for active participation. Both lectures and tutorials are typically held in-person on campus. For some years, however, alternative designs have been offered, which are described below.

### On-Campus vs Distance Courses

To refer to the traditional design of preparatory courses, we speak of ‘on-campus courses’ with ‘on-campus lectures’ and ‘on-campus tutorials’ since teaching involves direct interaction between teachers and students on campus. In contrast, for several years, courses have also been offered that take place completely or partly in the form of self-study (Derr et al., 2018; Fischer, 2014; Greefrath et al., 2017). Such online learning courses offer first-year students the opportunity to prepare for their studies without being located at the university itself. This not only reduces the barrier of commuting but also creates opportunities for those who study dual or part-time to participate. In addition, online learning courses enable the individualized use of materials, allowing first-year students to work at their own pace and intensity. To this end, interactive learning materials are provided via learning management systems that also enable communication between instructors and learners. We refer to these types of preparation courses as ‘(asynchronous) online learning courses’, which may include recorded ‘(asynchronous) online lectures’ but no tutorials. Combining features of on-campus and online courses results in ‘blended learning courses’. These often take place over a longer period of one to two months, in which self-regulated online learning alternates with classroom events on-site at different intervals (Fischer, 2014).

Pandemic-related restrictions further expanded the range of course options. By transforming on-campus courses into digital formats, albeit in a way that the courses are almost unchanged, types of courses have emerged that we call ‘(real-time or synchronous) distance courses’. These courses take place exclusively online but increasingly include synchronous elements in the form of video conferences. In this way, direct interactions between students and lecturers are made possible despite the distance. This applies in particular to the implementation of tutorials, which use breakout sessions and shared screens to implement classroom activities. We refer to this particular form of tutorials as ‘(real-time/synchronous) distance tutorials’. Since lectures are less interactive, both ‘(real-time/synchronous) distance lectures’ and ‘(asynchronous) online lectures’ are common, the latter being offered through video recordings, for example by broadcasting a traditional blackboard lecture or providing slides with audio commentary (Büchele et al., 2021; Kempen & Lankeit, 2021).

In this paper, we compare two specific variants of a preparatory course: a preparatory course with (synchronous) distance tutorials and (asynchronous) online lectures and a preparatory course with on-campus tutorials and the same (asynchronous) online lectures. In this way, these results provide helpful insight into the effectiveness of recent synchronous distance learning compared to traditional learning environments.

### Students’ Participation in Voluntary Preparatory Courses

If participation is voluntary, evaluation research in the context of preparatory courses often addresses the question of which students participate in which type of preparatory course. Overall, previous studies indicated that participants in preparatory courses are positively selected and that not all students who could benefit from such courses are reached (e.g. Büchele et al., 2022; Park et al., 2018; Voßkamp & Laging, 2014). For the German context, for example, Büchele et al. (2022) have shown that ‘students with […] worse prior GPA [grade point average, German *Abitur* grade] show significantly lower participation in the preparatory course’ (p. 14). Similarly, other studies have reported that first-year students who were less successful in demonstrating mathematical skills in high school class tests are generally less likely to choose a preparatory course than those students with higher test scores (Voßkamp & Laging, 2014).

Regarding the type of preparatory course, students with a lower grade point average at school tend to prefer on-campus courses, while students with higher grades tend to choose (asynchronous) online or blended learning courses (Derr et al., 2018; Fischer, 2014; Greefrath et al., 2017). In addition to prior knowledge, individual affective characteristics such as one's attitude toward online learning or an individual’s learning routines can influence course decision: Students who prefer self-regulated learning and have a more positive attitude towards computer use are more likely to choose (asynchronous) online or blended learning courses, while students in on-campus courses value personal contact and peer learning (Biehler et al., 2011; Greefrath et al., 2017). The trends in course decisions described here must be considered when comparing different types of courses.

### Performance Gains During a Preparatory Course

Various studies have shown that students can achieve performance gains during a preparatory course (Derr et al., 2018; Hoever & Greefrath, 2021). However, performance tests conducted at the beginning and end of preparatory courses often show a wide dispersion. Thus, the effect of preparatory courses must be discussed in a differentiated manner. For example, Derr et al. (2021) showed that students with lower scores in the pre-test achieved higher performance gains than their fellow students with higher entrance scores. Nevertheless, they could only partially compensate for the performance differences with their fellow students. The strongest increase in performance was observed among first-year students who had good mathematics grades or a high grade point average at school (German *Abitur* grade) but scored low on the pre-test (Derr et al., 2018).

The extent to which performance gains are achieved during the preparatory course also depends on factors such as the student’s engagement (Derr et al., 2021) and the chosen type of course. Yet, the results do not point in a clear direction. For example, some studies have indicated differences in favor of (asynchronous) online or blended learning courses (Ashby et al., 2011; Derr et al., 2018; Fischer, 2014; Greefrath et al., 2017), while others have reported differences in favor of on-campus courses (Dondorf et al., 2016; Francis et al., 2019). For example, a study of 1376 electrical engineering and computer science students at the University of Kassel, Germany, showed that those who chose an asynchronous online learning course performed significantly better in a subsequent mathematics exam than those who took an on-campus course (Greefrath et al., 2017). Similarly, Derr et al. (2018) and Fischer (2014) reported that students of different programs show a greater increase in performance when they participate in a distance or blended learning course. In contrast, a comparative study at the University of Aachen in which 130 students were randomly assigned to an asynchronous online course and an on-campus course showed that the on-campus group performed significantly better after working through the same content, while the distance learning group did not (Dondorf et al., 2016). However, and not specific to mathematics, there is evidence that synchronous distance courses can be just as effective as on-campus courses (Mullen, 2020). Consistent with this, initial studies comparing preparatory courses before and during the pandemic showed that distance courses with synchronous tutorials have lower participation rates but can be similarly effective as on-campus courses in terms of mathematic performance (Büchele et al., 2021).

## Affective Factors Influencing Course Performance

According to theories of person–environment fit (Etzel & Nagy, 2016; Tracey et al., 2012), successful learning depends on how well the individual characteristics of the learner match the characteristics of the learning environment. While a good fit of needs and offerings as well as prerequisites and requirements promotes academic success and well-being, an inadequate fit can lead to failure and demotivation. Since distance and on-campus courses differ in specific characteristics (e.g. Artino & Stephens, 2009; Derr et al., 2021; Michinov et al., 2011; Reinhold et al., 2021), they create learning environments with different offerings and requirements. Thus, it is conceivable that students may experience varying degrees of success in distance and on-campus courses, depending on their individual needs and prerequisites. Indeed, studies of the first pandemic semester have shown that students with different affective characteristics, such as self-efficacy, self-regulation, or motivation, differ in terms of well-being and academic success when working in distance learning environments (Händel et al., 2022; Kempen & Liebendörfer, 2021). Therefore, it is reasonable to consider affective factors of person-environment fit when comparing performance gains in distance and on-campus courses. In order to describe the fit between person and environment in a way that is as differentiated as possible, we distinguish between general and specific affective factors. While general affective factors are more person-related and have no direct relation to the characteristics of a learning environment, specific ones are more closely related to the characteristics of distance and on-campus learning. Accordingly, variation in course modality may directly affect person-environment fit, particularly via specific affective factors.

### General Affective Factors

#### Self-Efficacy

The concept of self-efficacy refers to the confidence in one’s own abilities to carry out certain actions successfully (Bandura, 1997). From a subject-related perspective, *mathematical* self-efficacy describes the confidence in one’s own ability to perform *mathematical* activities successfully (Pajares & Miller, 1994). This can refer to solving concrete tasks, proving mathematical statements or, more generally, coping with the study of mathematics.

Several studies have shown a relationship between students’ self-efficacy and their mathematics performance (Hackett & Betz, 1989; Jaafar & Ayub, 2010; Liu & Koirala, 2009). The influence of self-efficacy can be direct or indirect, with the indirect influence being moderated by, for example, interest, social familiarity, or metacognitive and self-regulatory strategies (Schunk & Pajares, 2002). However, a recent study by Kempen and Liebendörfer (2021) showed that students' self-efficacy was higher when they preferred elements of traditional on-campus learning over digital learning resources. Therefore, distance learning and self-efficacy could be negatively related.

#### Satisfaction

Satisfaction is considered a retrospective emotion with positive connotations and is based both on affective experiences and on cognitive comparisons between expectation and experience (Blüthmann, 2012; Li et al., 2013; Schiefele & Jacob-Ebbinghaus, 2006). Satisfaction and performance influence each other insofar as high performance and a feeling of achievement increase satisfaction (Balkis, 2013; Li et al., 2013; Scheunemann et al., 2021; Wiers-Jenssen et al., 2002). If satisfaction results from the fit between individual needs, attitudes, and abilities and the characteristics of the learning environment, it can be assumed that variation in course modality can influence satisfaction. For example, Händel et al. (2022) reported higher satisfaction among students when their learning requirements in terms of equipment and digital skills match the requirements of distance learning.

#### Students’ Engagement

Students’ engagement is described as the commitment of physical and mental energy that a student brings to the academic experience (Astin, 1984). It is closely related to learning outcomes (Baron & Corbin, 2012). As an indicator of student engagement, one can use the minimum condition for engagement, namely participation in tutorials (Derr et al., 2018). With respect to preparatory courses, Kürten (2020) reported effects of physical presence, finding that students who attend tutorials more frequently achieve higher mathematics scores at the end of a preparatory course. Students’ engagement may be even more important in distance courses, with dishonesty increasing in this course type (Büchele et al., 2021).

### Specific Affective Factors

#### Self-Regulation and Procrastination

Self-regulation is understood as ‘*proactive* processes that students use to acquire academic skill, such as setting goals, selecting and deploying strategies, and self-monitoring one’s effectiveness’ (Zimmerman, 2008, p. 166). Unlike at school, learning processes at university consist of many self-learning phases that place high demands on time management and process monitoring (Clark & Lovric, 2008; De Guzmán et al., 1998; Rach & Heinze, 2017). The high cognitive, time, and motivational load in the absence of external orienting features may reveal self-regulatory dysfunction, which manifests itself in procrastinating behavior (Vermunt & Vermetten, 2004). By (academic) procrastination, Solomon and Rothblum (1984) refer to ‘the act of needlessly delaying intended tasks to the point of experiencing subjective discomfort’ (p. 503). In a meta-analysis of 33 studies, Kim and Seo (2015) showed that procrastination and academic performance correlate strongly and negatively. The relationship between performance development and procrastination becomes particularly important in distance learning. Since there are hardly any external regulators, such as compulsory attendance at lectures, in distance learning, self-regulation gains importance (Artino & Stephens, 2009; Derr et al., 2021; Michinov et al., 2011; Reinhold et al., 2021). Comparing on-campus and distance learning, initial studies have indicated that students tend to procrastinate more in asynchronous online learning than in on-campus learning (Yilmaz, 2017). Consistent with this, mathematics students in a study by Radmehr and Goodchild (2022) reported difficulties with time management in the first pandemic semester.

#### Social Relatedness

Social relatedness is understood as the feeling of belonging and connection to a reference group (Deci & Ryan, 1985). Various studies have shown a positive correlation between social relatedness and students’ satisfaction with their studies (F. Zimmermann et al., 2018). Students who drop out of their studies at an early stage feel less socially integrated and report less cooperation with other students (Geisler, 2020). Against this backdrop, social relatedness can be considered an important factor in the transition from school to higher education. However, pandemic constraints have challenged any kind of networking. Accordingly, research reported that social integration in distance learning was difficult for many students (Marczuk et al., 2021; Radmehr & Goodchild, 2022). The use of videoconferencing has enabled comparatively communicative and cooperative work, but could only partially compensate for the social restrictions, especially in the area of informal exchanges (Händel et al., 2022; Kempen & Lankeit, 2021; Marczuk et al., 2021). Accordingly, attending a preparatory course in distance learning could also have a negative impact on students’ social relatedness, and thus indirectly on their performance.

#### Digital Readiness

Hong and Kim (2018) define *digital* or *online learning readiness* as ‘technology-related knowledge, skills, and attitudes and competencies for using digital technologies to meet educational aims and expectations in higher education’ (p. 304). In addition to general skills for self-regulated learning, digital readiness also includes internet and computer self-efficacy as well as online communication self-efficacy (Hong & Kim, 2018; Hung et al., 2010). Empirical findings on regular or pandemic online courses have suggested that students with a high digital readiness perform better in courses (Johnson et al., 2008; Keramati et al., 2011; Taşkin & Erzurumlu, 2021), report higher satisfaction (Johnson et al., 2008; Kuo et al., 2014), procrastinate less (Ergene & Türk Kurtça, 2020) and feel less lonely (Händel et al., 2022). In this sense, digital readiness could directly or indirectly influence mathematics performance in distance learning.

## Summary and Research Questions

The study presented here aims to compare the effectiveness of newly developed distance formats with classic on-campus offerings in the context of preparatory courses. Therefore, our research is guided by three research questions.

1.  How does mathematics performance change during the preparatory course, and what influence does the variant of tutorial have on it?

We expect participants from both tutorial variants to achieve performance gains during the preparatory course (we refer to this hypothesis as H1.1, and use similar numbering for other hypotheses). Although previous research is inconsistent regarding the influence of course modality (Ashby et al., 2011; Francis et al., 2019), studies by Greefrath et al. (2017) and Fischer (2014) have indicated that participants perform better at the end of the preparatory course if it is in an online or blended learning format. In line with the observations of Büchele et al. (2021), we expect that this also applies to (synchronous) distance learning. We therefore hypothesize that students in distance courses show equivalent or even stronger performance gains than their peers in on-campus courses (H1.2).

RQ2.  To what extent does prior knowledge as assessed in an entrance test influence participants’ performance gains and to what extent is this relationship moderated by tutorial variation?

Since prior knowledge that students can draw on during the course can strongly influence course performance (Derr et al., 2018; Lagerlöf & Seltzer, 2009), we assess students’ prior knowledge at the beginning of the course using a mathematics test. The results of the tests represent a snapshot of recallable knowledge at the time of the test. Nevertheless, we expect that the performance of students with higher and lower test scores will increase differently (H2.1). This effect can be additionally strengthened by the tutorial variant, since distance learning places additional demands on participants, such as self-regulation (Artino & Stephens, 2009; Michinov et al., 2011; Reinhold et al., 2021). Therefore, we expect that students who score lower on the entrance test will benefit more from on-campus courses (H2.2).

RQ3.  To what extent are affective factors (general and specific) predictive of performance gains in the different tutorial variants?

With reference to the outlined state of research, we assume that course performance is influenced by various affective factors. Since on-campus and distance courses place different demands on learning, it can be assumed that factors that directly affect the design of the learning environment are more strongly related to performance gains in that environment. Thus, specific affective factors such as procrastination, social relatedness, and digital readiness may have variant-specific effects. While procrastination and digital readiness are of particular relevance in distance learning from a theoretical perspective (Chung et al., 2022; Händel et al., 2022), social relatedness is a characteristic feature of on-campus teaching (Biehler et al., 2011) (H3.1). General affective factors such as self-efficacy, satisfaction, and engagement, on the other hand, are assumed to influence performance in both on-campus and distance learning contexts (H3.2). The relationship between the individual factors will be explored for both tutorial variants.

## Material and Method

### Design and Sample

The current study was conducted as part of an annual two-week preparatory course at the University of Münster. This course is designed for entering student teachers at the primary (grades 1–4) and lower secondary (grades 5–10) levels, who form a homogeneous group in that both study programs contain a comparable amount of mathematics and begin with lectures in arithmetic and geometry. Since for these two lectures prior knowledge from lower secondary school such as fractions, algebraic transformations or geometric theorems is particularly relevant, the preparatory course focuses on these contents. In general, sound knowledge and skills in (lower) secondary mathematics are a central prerequisite for a deeper understanding of the same and thus necessary for access to university mathematics (Deeken et al., 2020; Deng, 2007). In particular, student teachers who will later teach this content should address their challenges in these areas before beginning their studies.

The course traditionally consists of eight lectures and seven tutorial sessions. Each tutorial session is accompanied by tasks that encourage practice and application of the topics covered in the lecture. The aim of the preparatory course is both to deepen mathematical knowledge from school and introduce students to university mathematics. To this end, three lectures focus on reinforcing the basic mathematical skills in arithmetic, algebra, and geometry that will be relevant in the first-year lectures. Another three lectures cover aspects of mathematical thinking, such as formulating definitions, reading theorems, and proving mathematical statements. Again, tasks and examples were chosen from lower secondary school, but they became increasingly formalized as the course progressed. The remaining two lectures deal with self-regulation and learning strategies at university. Using set theory and divisibility theory as examples, strategies for elaboration and organization of mathematical knowledge are developed. With the content and objectives described here, the course design relates to the expected entry requirements at university level (Deeken et al., 2020) and draws on general design principles for preparatory courses (Biehler et al., 2011; Greefrath et al., 2017).

Due to pandemic constraints, in 2021 the course was offered in two variants. In both of these variants, lectures were video-recorded and provided asynchronously on the university’s learning platform. The tutorial sessions, on the other hand, were offered synchronously either on campus or via distance learning video conferencing. For each of the two tutorial options, parallel sessions were offered in two time slots, so that there were three distance and three on-campus tutorials on the same topic in the morning and afternoon, respectively. To keep all tutorials comparable, the tutors followed predefined course plans that specified the topics to be covered and the learning processes to be promoted. In particular, teaching methods, such as collaborative work or presenting results, were designed to be similar across on-campus and distance tutorials. For this purpose, the classic elements of classroom teaching, such as group work and writing on the blackboard, were translated to the digital learning environment with the help of breakout sessions, integrated whiteboards and split screens. Since four of the eight tutors led both an on-campus and a distance tutorial, all tutors were trained to use both types of instructional methods and made aware of parallels in implementation. A treatment check based on regular reports from the tutors showed that all tutorials were conducted in accordance with the course plan. Slight discrepancies arose because some students in distance tutorials needed technical support, for example, to share the screen, which caused delays, especially at the beginning of the course. In addition, tutors reported varying degrees of group work intensity in some places. While students in on-campus tutorials wore masks and had private conversations primarily after the tutorial, breakout sessions in distance learning provided the only opportunity for private exchanges, which sometimes caused distractions.

In this context, we investigated N = 159 students, of whom n = 133 were pursuing a primary teaching degree (127 female, 5 male, 1 did not specify) and n = 26 were pursuing a secondary teaching degree (16 female, 10 male). Since everyone perceived the pandemic situation differently, participants were free to choose which tutorial to attend, but were evenly distributed among the parallel sessions. While n = 71 students chose on-campus tutorials, n = 88 students attended distance tutorials. The reported sample size includes only those students from whom data are available at both measuring points. In particular, students who dropped out of the preparatory course early were not included in this sample. Because preparatory courses are voluntary, the reasons for dropping out can be manifold (Kürten, 2020; Street, 2010). For example, overwork, underutilization, or other obligations such as finding a place to live can lead to dropout, making it difficult to predict the development of students who only participated in the pre-test. In addition, dropout rates tend to be higher in online learning environments, again challenging their predictive value (Ashby et al., 2011; Derr, 2017).

**Fig. 1:**
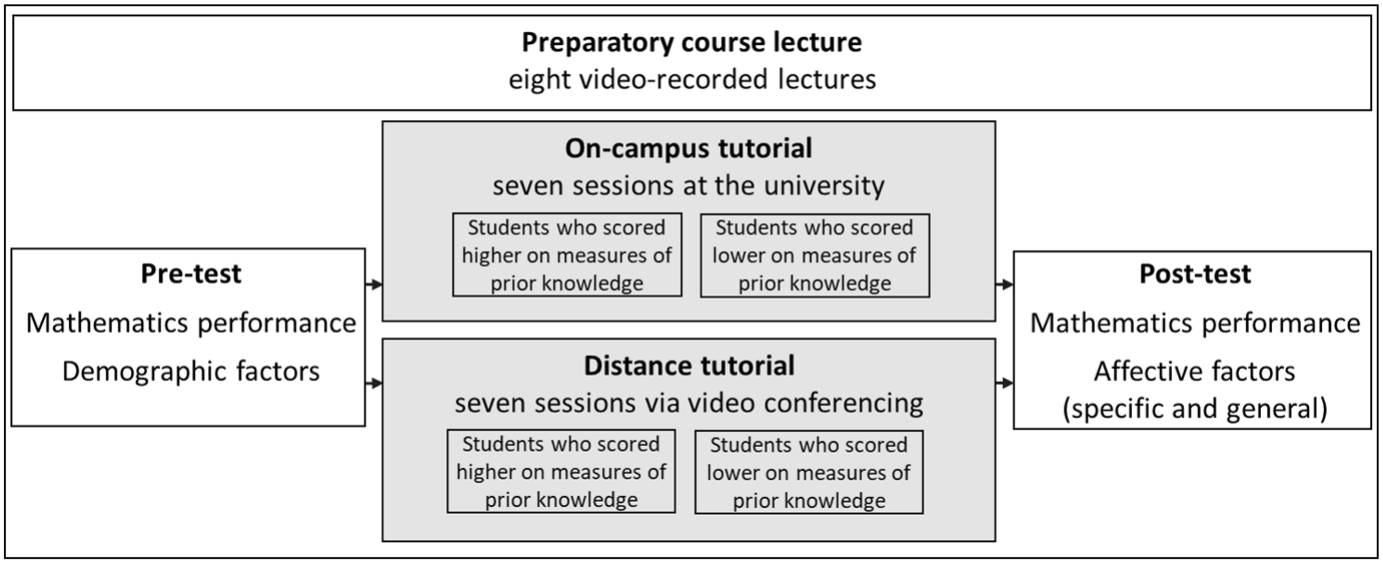
Overview of the study design

To address the research questions, the study follows a within-between-subject design (see Fig. 1). Whereas the students’ choice of tutorial variant served as a between-subject factor, their mathematics performance served as a within-subject factor. Mathematical performance was measured using a rotating test design with two measuring points, namely at the beginning of the first tutorial session and at the end of the final one. The pre-test also served to ascertain the students’ prior mathematical knowledge. Test administration was guided and monitored (on-site or via videoconference) by the respective tutors. For students in distance tutorials, a digital submission was set up. Tasks that were solved by handwriting were to be photographed by these students and submitted via an online platform.

In line with RQ3, we additionally measured students’ affective characteristics (general and specific) at the end of the preparatory course. Data on students’ demographic characteristics were collected at the first measuring point by asking them for their gender, age, study program, and grade point average at school (their *Abitur* grade).

### Instruments

#### Mathematics Performance

**Fig. 2:**
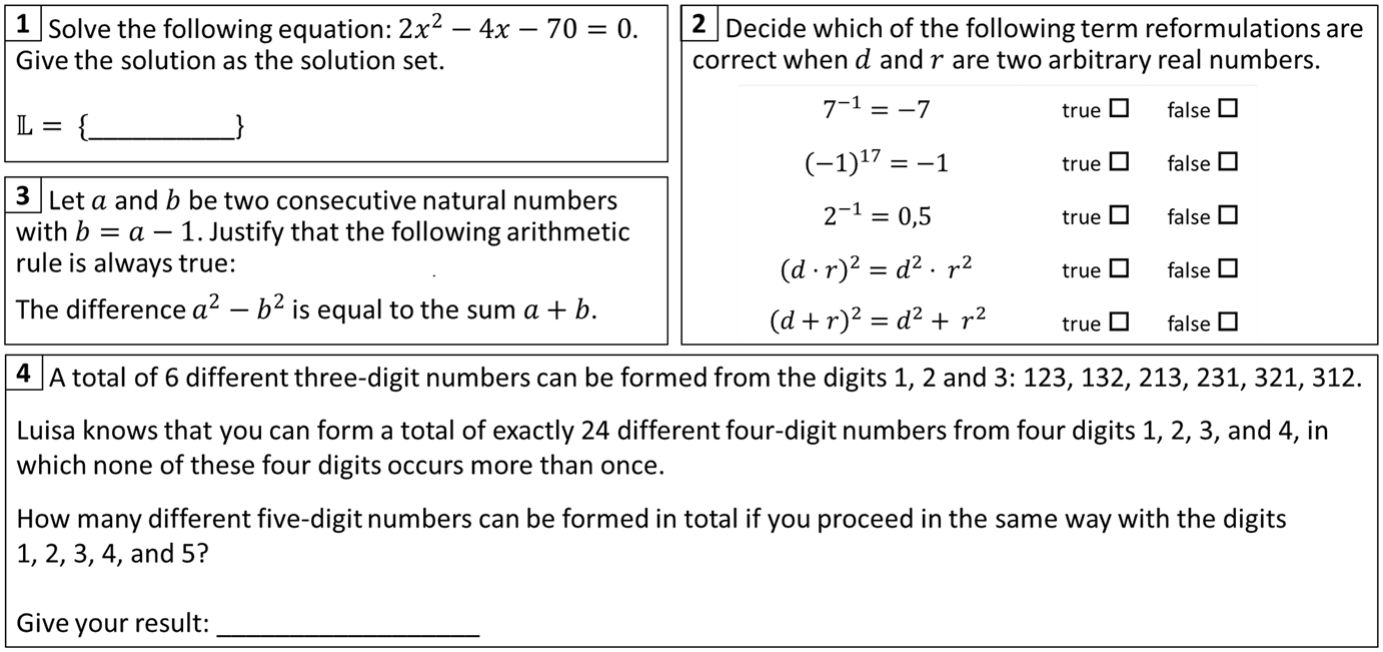
Sample items for measuring mathematics performance

To measure students’ mathematical performance, we used a total of 20 items, covering fractions, algebraic transformations, linear and quadratic functions, and geometric theorems, as these were the relevant topics of the preparatory course (see Section “Design and Sample”). The pool included both items that would test technical procedural knowledge (see Examples 1 & 2 in Fig. 2) and those that would promote exploration and require competencies in problem solving, reasoning, and argumentation (see Examples 3 & 4 in Fig. 2). The more technically oriented items came from TIMSS/II for the 7th/8th grade (Baumert et al., 1998) or were self-developed. The items measuring problem solving and reasoning were retrieved from the Institute for Educational Quality Improvement (Blum et al., 2010; IQB, 2018) and are sample items for the written competency-based comparison tests that students take in the 8th grade in Germany.

Consistent with the rotating test design, we designed four test booklets (pre-distance, pre-campus, post-distance, post-campus) with 10 items each that were linked by common anchor items. For each item, students’ solutions were dichotomously coded as correct (1) or incorrect (0). Missing solutions were attributed to a lack of motivation or a lack of ability and were therefore scored as incorrect (Ludlow & O’leary, 1999; Mislevy & Wu, 1996). The empirical data from two measuring points were scaled using the one-parameter IRT Rasch model. The Rasch model uses a logit model that models the probability of a correct/incorrect answer as a logistic function of item difficulty and person ability. This allows item difficulties and personal characteristics to be located on a common scale, with the unit of measure being logit rather than summed scores. In the current study, the Rasch model also provided a means for assessing students’ abilities independent of the particular items that they worked on. For modelling purposes, we first considered the data from both measuring points together and determined the item parameters based on the ‘virtual person’ approach (Hartig & Kühnbach, 2006; Wang et al., 2004; B. D. Wright, 2003). In this approach, the data matrix is sorted by items only, so that scores from the same person at different measuring points are considered as scores from two (virtual) persons. The goodness of fit of the model was tested globally using the Andersen likelihood ratio test ($p >.05$) and locally using the Wald test ($p >.05$ for all items). An examination of the item fit values for each item revealed one item with an excessively high infit value (1.8), low item-total correlation (.09), and solution frequency (1.07%). This item, the content of which was a transfer of the Pythagorean theorem to equilateral triangles, was therefore excluded. After excluding this item, the weighted mean squares, with values ranging from .93 to 1.08, were within the desired limits of .8 to 1.2 (Bond & Fox, 2007). The item-total correlations ranged from .36 to .60 and thus could be considered satisfactory. The solution frequencies of the individual items varied between 14 and 93% with a mean value of 49.72%, indicating that a wide range of difficulty was covered.

To estimate the person parameters, the determined item parameters were exported and the data matrix was restructured according to the ‘virtual item’ approach (Hartig & Kühnbach, 2006; B. D. Wright, 2003). The data series are now sorted by person to distinguish between pre-test and post-test. By using a two-dimensional model for scaling, two ability estimators, one for the pre-test and one for the post-test, were obtained for each person (Wang et al., 2004). The individual scales had an EAP/PV reliability of .633 at pre-test and .657 at post-test. Since the EAP/PV reliability compares the expected variance with the actual variance, it is comparable with Cronbach’s alpha. Therefore, the values obtained could be considered sufficient for group comparisons.

#### Affective Factors

For measuring *affective factors,* we made use of existing instruments with proven validity in previous studies. Overall, we collected data on three *general* affective factors, namely mathematical self-efficacy, satisfaction, and engagement, and on three *specific* affective factors, namely procrastination, social relatedness, and digital readiness. All these data were collected at the end of the preparatory course. Table 1 provides an overview of the characteristic values of the individual scales.

**Table 1:** Overview of the characteristic values of the individual scales

Predictor	Sample item	Value	# Items	α
Self-efficacy	‘In math, I am confident that I can understand even the most difficult material.’	1 to 4	4	.76
Satisfaction	‘I would recommend the preparatory course to everyone.’	1 to 4	3	.75
Engagement	‘Please indicate how many tutorial sessions you have attended.’	1 to 7	1	-
Procrastination	‘I don’t start an important task until I’m under pressure.’	1 to 7	7	.94
Social relatedness	‘In the preparatory course, I felt like I belonged.’	1 to 6	7	.81
Digital readiness	‘I find it easy to follow courses in a video conference.’	1 to 4	7	.68

*Mathematical self-efficacy* has been shown to be a meaningful construct in the analysis of preparatory courses and was assessed in this study using the scale from the WiGeMath project (Biehler et al., 2018; Hochmuth et al., 2018). This scale refers to general, not task-specific, mathematical self-efficacy, and thus measures students’ general confidence in their own mathematical abilities. To measure students’ *satisfaction* with the preparatory course, we asked the participants to compare their expectations and needs with the actual learning opportunities offered. For this purpose, we used an adapted version of the Satisfaction with Study Content Scale (ZSI) by Schiefele and Jacob-Ebbinghaus (2006). As an indicator of students’ *engagement*, we used their attendance in tutorials. Following previous studies (Derr et al., 2018; Eichler & Gradwohl, 2021; Fischer, 2014), we asked in a single item how many of the total seven tutorials the students had attended. Thus, this measure is limited to physical aspects and serves as a basic indicator for engagement (see discussion in Section “Affective Predictors of Mathematics Performance (RQ3)”).

Students’ *procrastination* was measured using the General Procrastination Questionnaire (APROF; Höcker et al., 2021). This scale asks about students’ general learning behavior and, in particular, the avoidance strategies they used during the preparatory course based on self-assessments. The APROF scale specifically focuses on task avoidance, time management, and alternative preferences and thus captures disturbances in self-regulation that have been shown to be relevant for college and university students (Johns, 2020; Solomon & Rothblum, 1984; Steel, 2007). To assess the *social relatedness* of students, two aspects of social interaction in the preparatory course were considered, following the WiGeMath project (Hochmuth et al., 2018), namely the students’ general sense of belonging (Rakoczy et al., 2005) and the formation of learning groups (LimST scale; Liebendörfer et al., 2021). Since working in a digital learning environment involves a variety of actions, *digital readiness* included different facets, such as application usage or information sharing. In order to reflect the requirements of attending distance tutorials as accurately as possible, we developed items for this scale ourselves using the digital readiness for academic engagement scale as our basis (DRAE; Hong & Kim, 2018). The developed scale covered a wide range of digital interaction and self-organization, such as collaborative work in video conferences and retrieving, sharing, and filing materials from the tutorials. The reliability of the six scales ranged from acceptable to excellent (see Table 1), with digital readiness having the lowest reliability (α = .68). Considering the broad scope of this construct, reliability could be considered satisfactory.

## Results

### Preliminary Analyses

Because the students in this study could self-assign to distance or on-campus tutorials, there was no random sample. To examine the comparability of the two treatment groups, we checked the group composition for disproportionate distributions in terms of demographic factors and entry prerequisites of the students using descriptive data and unpaired t-tests. The latter assumes a normal distribution of measures and homogeneity of error variances. While Levene’s tests showed that the variances were homogeneous for all relevant measures (p > .05), Kolmogorov–Smirnov tests indicated that the values for some measures were not normally distributed. However, given the sample size, we could assume that the parametric tests were robust and could still be applied (Rasch & Guiard, 2004).

Descriptive data showed that with respect to age (M_OC_ = 19.53, SD = 2.16 and M_D_ = 19.39, SD = 2.01), study program (80.3% and 86.4% primary level), and gender (88,7% and 92,0% female), an even distribution was achieved between on-campus and distance learners. In terms of performance-related characteristics, the two groups did not differ statistically significantly in either their grade point average at school (M_OC_ = 1.93, SD = .49 and M_D_ = 1.94, SD = .45; $t\left(154\right)=-0.13$, $p=.899$) nor their prior mathematical knowledge as assigned in the pre-test (M_OC_ = -0.35, SD = 1.32 and M_D_ = -0.32, SD = 1.14, $t\left(157\right)=-0.16$, $p=.876$). In addition, students reported comparable engagement in lectures by working through an average of six lecture videos (M_OC_ = 6.17, SD = 0.99 and M_D_ 6.44, SD = 1.12, $t\left(144\right)=-1.52$, $p=.121$). Since RQ3 investigates the relationship between mathematics performance and affects, it was also worthwhile analyzing the composition of the sample in terms of general and specific affective characteristics (see Table 2).

**Table 2:** Means (and standard deviations) of students’ affective characteristics broken down by the type of tutorial attended

	**On-campus**	**Distance**	**Significance**
Self-efficacy	2.72 (0.39)	2.52 (0.45)	$t\left(156\right)=2.99,\,p=.003,\,d=0.48$
Satisfaction	3.66 (0.35)	3.46 (0.39)	$t\left(139\right)=3.27,\,p <.001,\,d=0.55$
Engagement	6.64 (0.66)	6.55 (0.94)	$t\left(144\right)=0.68,\,p=.499,\,d=0.11$
Procrastination	3.06 (1.05)	2.94 (1.12)	$t\left(156\right)=0.69,\,p=.493,\,d=0.11$
Social relatedness	4.95 (0.88)	4.95 (0.62)	$t\left(156\right)=-0.06,\,p=.949,\,d=0.01$
Digital readiness	3.02 (0.47)	3.10 (0.37)	$t\left(156\right)=-1.20,\,p=.232,\,d=0.19$

T-tests showed that the two treatment groups differed statistically significantly in the characteristics of self-efficacy and satisfaction. Students in on-campus tutorials each had higher values than their peers in distance tutorials. This kind of sample composition should be considered when interpreting the results.

To answer our research questions, besides t-tests, we used multiple linear regressions. This statistical testing procedure allows us to determine the influence of a predictor on student performance gains while controlling for other influences by integrating covariates (e.g. Field, 2018; D. B. Wright, 2006). However, it imposes certain conditions on the data. For all performed regressions, we checked the assumptions of homoscedasticity, linearity, and normality of residuals using graphical information (plots of standardized predicted values against standardized residuals and partial plots of mathematical performance against the individual predictors and histograms). A closer inspection of the student residuals (-3 < SDR < 3) and Cook’s distance values (COO < .61) showed that there were no systematic outliers or influencing cases in the distributions. For each model, correlations between predictor variables were low (VIF < 2.09, tolerance > 4.08), indicating that multicollinearity was not a confounding factor in the analysis.

### Performance Gains in On-Campus and Distance Tutorials

Both RQ1 and RQ2 asked about the effectiveness of the preparatory course in terms of learning progress. Therefore, the focus was on students’ mathematical performance before and after attending the preparatory course. Table 3 shows the means (and standard deviations) of the students at both measuring points. Note that the unit of measure is logit and ranges from -4.41 to 4.02 in our data set. For a better interpretation of the values, Table 3 also shows the proportions of tasks solved correctly on average in square brackets. On average, mathematical performance in both tutorial variants improved significantly from the pre-test to the post-test. The differences (post–pre), 0.72 and 0.71, were statistically significant and represented medium effects.

**Table 3:** Means (and standard deviations) of students’ mathematical performance at pre- and post-test [and proportions of tasks solved correctly on average]

	**N**	**M (SD)**		**Significance**			
		pre	post				
On-campus	71	-0.35 (1.32) [38,9%]	0.37 (1.42) [55,6%]	$t\left(70\right)=-5.37,\,p<.001,\,d=-0.64$			
Distance	88	-0.32 (1.14) [41,8%]	0.39 (1.14) [57,4%]	$t\left(87\right)=-5.04,\,p<.001,\,d=-0.54$			

To analyze the effects of tutorial variation (RQ1) and retrievable prior knowledge (RQ2), we conducted a three-step linear regression with the gain scores (post–pre) as the dependent variable. Model 1 included only the tutorial variant as an independent variable, which was not found to predict students’ performance gains ($\beta =-.003,\,p=.966$). Model 1 also was no significant fit to the data (${R}^{2}=.000,\,p=.966$), indicating that choosing an on-campus or distance tutorial did not affect students’ learning progress (Table 4).

**Table 4:** Coefficients of the regression models (method: inclusion) predicting students’ gain scores (post–pre)

	**Model 1**			**Model 2a**			**Model 2b**		
Predictor	b	SE	β	b	SE	β	b	SE	β
Tutorial	-0.01	0.20	-.003	0.01	0.18	.002	-0.13	0.18	-.05
Prior knowledge				-0.48	0.07	-.47***	0.11	0.22	.10
Tutorial x prior knowledge							-0.40	0.14	-.61**
R^2^ (adj.)	.00			.22 (.21)			.26 (.25)		

*N* = 159; ^+^*p* < .1; *p < .05; **p <.01; ***p < .001

**Fig. 3:**
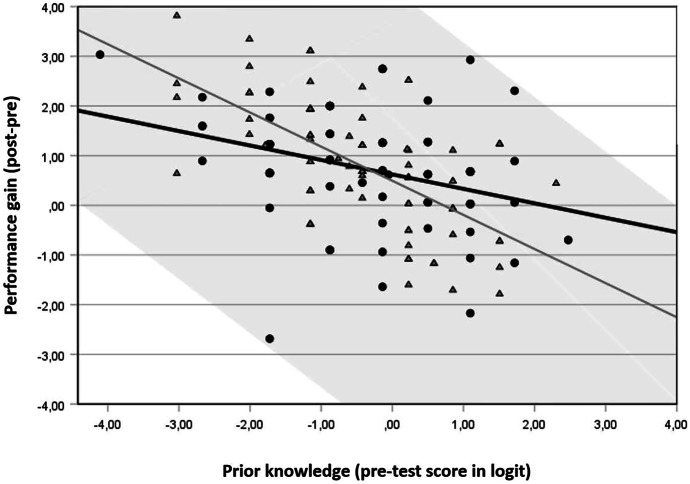
Simple slopes equations of the regression of performance gains on prior knowledge in on-campus (black, circles) and distance tutorials (gray, triangles); the range of possible value combinations is gray marked

By adding the prior knowledge assessed by the pre-test to the model, Model 2a significantly improved our ability to predict learning outcomes and explained 22% of the variance in performance gains (${R}^{2}=.22,\,p<.001$). In this model, the level of prior knowledge has a statistically significant negative impact on performance gains ($\beta =-.47,\,p<.001$). The beta value indicated that two students whose scores differed by one logit on the pre-test differed by only .47 logit on the post-test. Accordingly, the differences in performance between students with higher and lower scores on the pre-test remained, but decreased during the preparatory course. In Model 2b, the interaction of tutorial variation and prior knowledge was included as a third variable to account for moderation effects. Since the interaction was a statistically significant predictor ($\beta =-.61,\,p<.006$), we predicted that students with different levels of prior knowledge showed different performance gains, depending on which variant of the tutorial they chose. Figure 3 uses simple slopes to illustrate how tutorial variation influenced the relationship between prior knowledge and performance gains. Students with lower scores on the pre-test achieved higher gain scores in distance tutorials, while students with higher scores on the pre-test performed better in on-campus tutorials.

For more detailed analyses, we divided the sample based on the empirical mean into groups of students with above-average scores on measures of prior knowledge ($$n=79$$) and students with below-average scores on these measures ($$n=80$$). Separate regression analyses for both groups confirmed a statistically significant impact of tutorial variation for students demonstrating a higher level of prior knowledge ($$\beta =-.23$$, $$p=.041$$, $${R}^{2}=.053)$$. While students in on-campus tutorials significantly increased their mathematical performance by an average of 0.49 logit ($${\mathrm{M}}_{\mathrm{pre}}=0.63,\mathrm{ SD}= 0.69\;\mathrm{ and }\;{\mathrm{M}}_{\mathrm{post}}=1.12,\,\mathrm{ SD}=1.22,\,t\left(38\right)=2.63$$, $$p=.006$$, $$d=0.42)$$, those who chose distance tutorials actually performed 0.1 logit worse in the post-test than in the pre-test ($${\mathrm{M}}_{\mathrm{pre}}=0.68,\,\mathrm{ SD}= 0.55\;\mathrm{ and }\;{\mathrm{M}}_{\mathrm{post}}=0.59,\,\mathrm{ SD}=1.34$$). However, the decline in performance in this case was not statistically significant ($$t\left(39\right)=-0.42$$, $$p=.34,\,d=0.07)$$. For students demonstrating a lower level of prior knowledge, the analysis showed that the tutorial variant had no significant influence on students’ performance gains ($$\beta =.17, p=.122$$, $${R}^{2}=.030)$$.

### Affective Predictors of Mathematics Performance

To answer RQ3, multiple linear regressions were performed with gain scores (post–pre) as the dependent variable. To detect group differences, the regression models were calculated separately for the two conditions. In each case, a baseline model that included only the level of prior knowledge served as a reference model to control for the effects of pre-test scores. Since general affective factors are considered fundamental to successful learning regardless of the specific learning environment, these factors were included in the regression model in the first step. Model 3a thus included the three general affective factors as possible predictors and the prior knowledge as a covariate. For Model 3b, the three specific affective factors were added, which were more closely tied to the learning environment from a theoretical perspective. Table 5 summarizes the results for the on-campus tutorials and Table 6 for the distance tutorials. The F-tests showed that all models were a significant fit to the data ($p <.001$).

**Table 5:** Coefficients of the regression models (method: inclusion) predicting students’ performance gains for on-campus tutorials

		**Model 3a**			**Model 3b**		
	Predictor	b	SE	β	b SE	SE	β
On-campus	Prior knowledge	-0.35	0.10	-.41***	-0.37	0.09	-.42***
	Self-efficacy	0.91	0.35	.31*	0.84	0.34	.28*
	Satisfaction	-.32	0.40	-.10	-0.22	0.50	-.07
	Engagement	0.52	0.20	.30*	0.66	0.10	.38**
	Procrastination				0.29	0.13	.26*
	Social relatedness				-0.12	0.19	-.09
	Digital readiness				0.17	0.27	.07
	R^2^ (adj.)	.28 (.23)			.35 (.27)		

*N* = 159; ^+^*p* < .1; *p < .05; **p < .01; ***p < .001

**Table 6:** Coefficients of the regression models (method: inclusion) predicting students’ performance gains for distance tutorials

		**Model 3a**			**Model 3b**		
	Predictor	b	SE	β	b	SE	β
Distance	Prior knowledge	-0.68	0.10	-.64**	-0.69	0.09	-.65**
	Self-efficacy	0.73	0.27	.26**	0.63	0.26	.22*
	Satisfaction	0.56	0.30	.18^+^	0.82	0.31	.26*
	Engagement	0.11	0.12	.08	0.12	0.11	.09
	Procrastination				-0.20	0.09	-.19*
	Social relatedness				-0.46	0.17	-.24**
	Digital readiness				-0.19	0.31	-.06
	R^2^ (adj.)	.48 (.45)			.56 (.52)		

*N* = 159; ^+^*p* < .1; *p < .05; **p < .01; ***p < .001

The inclusion of general affective factors in the baseline model resulted in Model 3a explaining 28% and 48% of the variance in performance gains, each representing a significant increase from the baseline model ($\Delta {R}_{OC}^{2}=.17,\,p=.004;\;\Delta {R}_{D}^{2}=.14,\,p <.001$). For both on-campus and distance tutorials, mathematical self-efficacy was a statistically significant positive predictor ($\beta =.31,\,p=.011$ and $\beta =.26,\,p=.009$). Beta values indicated that students’ gain scores increased by .91 and .73 logit, respectively, when self-efficacy increased by one point on the Likert scale. However, student satisfaction and engagement predicted performance scores differently in the two conditions. While satisfaction had a weakly significant impact in the distance tutorials ($\beta =.18,\,p=.070$), student engagement was positively related to learning progress only in the on-campus tutorials ($\beta =.30,\,p=.010$). Overall, controlling for students’ pre-test scores, Model 3a suggested that students in on-campus tutorials achieved better learning outcomes when they felt self-efficient and attended tutorials regularly. Students in distance tutorials, on the other hand, performed better when they felt self-efficient and were satisfied with the offered learning environment. The standardized beta values indicated that the two relevant affective variables each had a comparable (small) effect.

**Fig. 4:**
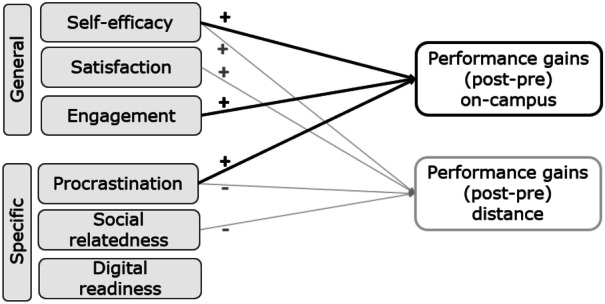
Overview of positive and negative predictors of performance gains in on-campus (black) and distance (gray) tutorials, controlled for prior knowledge

Model 3b, in addition, contained specific affective factors that were theoretically related to characteristics of on-campus or distance learning environments. Comparing the portion of explained variance for both tutorial variants, the analyses indicated a different weighting of the general and specific factors. While Model 3b for distance tutorials explained an additional 8% of the variation in mathematics performance gains ($p=.007$), there was no statistically significant improvement from Model 3a to Model 3b for on-campus tutorials ($\Delta {R}_{OC}^{2}=.07,\,p=.103$). Overall, Model 3b explained 35% of the variance in performance gains in on-campus tutorials and 56% in distance tutorials. Of the three variables entered into Model 3b, only procrastination significantly contributed to the prediction of performance gains in both conditions. However, the direction of the relationship was the opposite: while procrastination was a positive predictor in on-campus tutorials ($\beta =.26,\,p=.029)$, it was negatively related to mathematics performance gains in distance tutorials ($\beta =-.19\,p=.028)$. For distance tutorials, Model 3b also revealed a negative relationship with a small effect between students’ performance gains and their social relatedness ($\beta =.26,\,p=.008).$ The beta value indicated that as social relatedness increased by one point on the Likert scale, students’ mathematics performance decreased by .46 logit. Regardless of the type of tutorial, digital relatedness had no additional impact on performance gains. Figure 4 summarizes the results by depicting the relevant (positive or negative) predictors as arrows.

## Discussion

Since preparatory courses are—at least in Germany—a widely used approach to improve students’ study prerequisites, the present study questions the effectiveness of different course variants. In particular, we focus on the course’s modality, as person–environment fit theories indicate that the modality of the course may influence the learning process and thus the learning outcome. Consequently, the study examines the extent to which on-campus and (synchronous) distance tutorials meet students’ needs and lead to performance gains.

### Performance Gains in On-Campus and Distance Tutorials (RQ1 & RQ2)

First of all, the results of this study confirm that performance gains can be observed even in a two-week preparatory course. On average, the participants significantly improved their mathematical skills. Hypothesis H1.1 can thus be confirmed, and at least a short-term effect of preparatory courses can be demonstrated as in previous studies (Büchele, 2020; Derr et al., 2021; Hoever & Greefrath, 2021). Nonetheless, it should be noted that the preparatory course studied mainly repeated competencies from lower secondary level. Although sound knowledge and skills in lower secondary mathematics are critical for entering university mathematics, especially in teacher education programs (Deeken et al., 2020; Deng, 2007), follow-up studies may also consider other elements of knowledge.

In analyzing the impact of tutorial variation, we found no differences in performance gains between students in on-campus and distance tutorials, which is consistent with the hypothesis of equivalent development (H1.2). Since both groups of students increased their mathematics performance comparably on average, the chosen preparatory course variant is not a decisive influencing factor here. This result differs from previous studies reporting differences in favor of online courses (Ashby et al., 2011; Derr et al., 2018; Greefrath et al., 2017) or on-campus courses (Dondorf et al., 2016; Francis et al., 2019). However, unlike these studies, in this study, we compared two synchronous course designs that followed similar course plans. Thus, the divergent results could be explained by the different designs. This would suggest that the specific course design (asynchronous vs synchronous) and associated requirements influence course performance more than the modality of the learning environment (digital vs analog) per se. However, the results reported here must be interpreted with caution in that selection effects or dishonest responses may confound the results. Although on-campus and distance students did not differ in key characteristics such as age, study program, prior GPA, and pre-test score, biases may occur due to the free choice of a treatment condition. Furthermore, Büchele et al. (2021) reported cheating tendencies in distance learning, which cannot be ruled out in this study either, despite digital supervision. In addition, no statement can be made about whether differences only become apparent when the course takes longer, as is the case in other preparatory or regular mathematics courses.

Considering the *prior knowledge* of the students, differences in course performance become apparent. Regardless of the tutorial variant chosen, individuals who scored lower on the prior knowledge assessment showed greater performance gains, which confirms hypothesis 2.1. Accordingly, differences in entry prerequisites can be reduced by the preparatory course. However, despite their high performance gains, these students cannot compensate for differences from students who scored higher on the prior knowledge assessment. Similar results have also been reported in previous studies (Derr et al., 2018). Since preparatory courses aim to strengthen the prerequisite skills of entering students and reduce deficits, the observed progression is desirable but could be even stronger.

Additionally, we were able to demonstrate a moderation effect in our sample, according to which the *relationship between prior knowledge and performance gains* is influenced by the tutorial variant chosen. While students with lower scores on the prior knowledge assessment showed comparable performance gains in both on-campus and distance tutorials, the choice of tutorial variant emerged as a decisive factor for students with higher scores on this test. In particular, only students in the on-campus tutorials increased their mathematics performance. Hypothesis 2.2 is thus not confirmed and must be revised to the effect that students who demonstrated higher prior knowledge at the beginning of the course benefit more from on-campus tutorials. Since, according to the tutors, the breakout sessions were also used for non-mathematical discussions, a possible explanation for this finding could be increased cognitive activation during on-campus tutoring. Students with higher proficiency levels, and therefore lower motivation to learn, might tend to wander off during distance tutorials and participate with less concentration and focus. In on-campus tutorials, however, participation inevitably involves interaction. Exchanges with fellow students can raise further questions, or mutual explanations lead to a deeper understanding. The aspects described here should be considered in the design of preparatory courses, such as by encouraging even more exchange among students in distance learning in order to increase cognitive activation and commitment.

However, it is important to bear in mind that the selected sample does not allow direct conclusions to be drawn about mathematics courses in general. Since participation in the preparatory course was voluntary and not all students do participate in such offers (Büchele et al., 2022; Voßkamp & Laging, 2014), conclusions about the entire student body are only possible to a limited extent. Nevertheless, since the preparatory course conducted here has many overlaps with courses at other universities in terms of topic selection and design (Biehler et al., 2011; Greefrath et al., 2017), the implications may also be worth discussing for preparatory courses with other target groups.

### Affective Predictors of Mathematics Performance (RQ3)

Investigating the effectiveness of preparatory courses involves examining not only mathematics performance but also the factors that promote or inhibit learning processes in distance and on-campus tutorials. When looking at the general affective factors, only *self-efficacy* proved to be a positive predictor of mathematics performance in both on-campus and distance learning. Nevertheless, *satisfaction* and *engagement* were positively related to mathematics performance gains, with satisfaction being a relevant predictor for distance learning and engagement for on-campus learning. Hypothesis 3.2, according to which all general affective factors predict mathematics performance gains during the preparatory course, can thus only be partially confirmed. That self-efficacy positively influences course performance has been widely reported (Hackett & Betz, 1989; Jaafar & Ayub, 2010; Liu & Koirala, 2009). Although research findings on pandemic distance learning have questioned the strength of self-efficacy (e.g. Kempen & Liebendörfer, 2021), the results of this study also confirm the predictive relationship for distance learning. Furthermore, a positive relationship between satisfaction and performance, which has been previously reported for other course designs (Balkis, 2013; Li et al., 2013; Scheunemann et al., 2021; Wiers-Jenssen et al., 2002), was demonstrated for the first time for synchronous distance learning in this study. The weak influence of satisfaction in on-campus tutorials may be explained by the fact that this group of students was, on average, significantly more satisfied with the preparatory course than their peers. In addition to ceiling effects, student satisfaction here is presumably also shaped by side-effects, such as getting to know the university campus and making initial contacts. In this respect, the satisfaction measure for distance tutorials is probably more strongly linked to the actual content of the preparatory course. Similarly, the stronger relationship between engagement and on-campus learning could be explained by the instrument chosen. The measurement of engagement in this study was limited to attendance as a basic indicator. Whereas physical attendance at on-campus courses is usually accompanied by a minimum of active participation, and exchanges with fellow students often continue afterward, this is not necessarily the case with distance learning. Here, active participation and self-study before and after the course are of greater importance. In future studies, therefore, the degree of active participation or the extent of self-study could be recorded in addition to attendance.

Regarding the specific affective factors, procrastination and digital readiness were expected to predict mathematics performance for distance tutorials, whereas social relatedness was primarily considered to be a relevant influencing factor in on-campus tutorials (H3.1). However, the results of the studies do not confirm this hypothesis but shift the focus to other relationships. *Procrastination* proved to be a negative predictor of performance gains in distance tutorials in this study, which is consistent with previous findings (Artino & Stephens, 2009; Derr et al., 2021; Michinov et al., 2011; Reinhold et al., 2021; Romano et al., 2005; Yilmaz, 2017). However, procrastination in on-campus courses was positively related to mathematics performance gains. When interpreting this result, it should be considered that there were fixed attendance times for both distance and on-campus tutorials during the preparatory course. Therefore, the procrastination measure mainly refers to the preparation and repetition of the tutorial material. However, a lack of self-study can possibly be compensated by active participation in a two-week preparatory course. Future studies should therefore investigate the extent to which selection effects rather than performance effects are presented here, that is, that students with a tendency towards procrastination are more likely to choose an on-campus course. *Social relatedness* emerges as a negative predictor of distance learning in this study. Since social relatedness in the sense of belonging and peer learning is considered characteristic of on-campus learning (Biehler et al., 2011), this finding is surprising but may be explained by different socialization opportunities in both tutorial variants (see “Design and Sample”). Based on tutors' reports, it is possible that students in distance tutorials are more likely to use collaboration time to chat than to discuss mathematics. Finally, the results of this study suggest that there is no relationship between *digital readiness* and mathematics performance, which is also surprising given the research on pandemic distance learning (Chung et al., 2022; Händel et al., 2022). However, the first-year students in the 2021 preparatory course had gone through several phases of pandemic-driven distance education in their school careers. During this time, they may have developed sufficient skills in digital interaction and self-organization so that digital readiness may no longer be critical for a gainful use of the learning environment.

In general, the results show that the proportion of variance in performance gain is significantly increased by entering specific affective factors only in the distance tutorials. However, in both tutorial variants, prior knowledge had the strongest impact on predicting performance gains. Moreover, other possible influencing factors remain unaccounted for. Although Model 3b explains 35% and 56% of the variance in student performance gains, it remains unknown which further factors could explain parts of the remaining variance. In addition to the degree of active participation already mentioned, examining learning strategies could be a purposeful approach.

## Conclusion

Overall, our findings indicate that distance learning with synchronous elements represents a learning environment with its own characteristics. In summary, students who participate in on-campus tutorials achieve higher performance gains during a preparation course—even if they tend to procrastinate—if they have high self-efficacy and attend tutorials regularly. In contrast, students who participate in distance tutorials are more successful if they have high self-efficacy, are satisfied with the course offered, seldom procrastinate, and socialize less during tutorial sessions. These findings should be considered when planning on-campus and distance preparatory courses in order to best support learning in both variants. For example, spaces for informal exchange could be created in distance tutorials to increase opportunities for socialization outside the regular program.

Independent of affective factors, the results of this study show that preparatory courses with on-campus and distance tutorials can be similarly effective under certain conditions. The results therefore suggest that in the future, preparatory courses should be offered with distance and on-campus courses in parallel. Unlike distance-only self-paced courses, such an offering does not require a separate course design but provides an equivalent alternative for students who are unable to attend on-campus due to their location or other commitments. Particular attention should be paid to integrating learning opportunities for high achievers into distance learning.

Although the preparatory course studied differs from regular credit-bearing mathematics courses, it has partly similar framework conditions, such as the division into lectures and tutorials. Therefore, the reported influences of prior knowledge and affective factors should also be discussed in the design of other undergraduate courses that want to take advantage of the potential of synchronous distance learning even after the pandemic.
